# Trends and Disparities in Mortality Due to Gastric Malignancies the United States: A Nationwide Analysis from 1999 to 2020

**DOI:** 10.1007/s12029-025-01295-9

**Published:** 2025-08-05

**Authors:** Muhammad Ahmad, Aizaz Ali, Tahreem Mari, Fariha Hasan, Saeed Ali, Mohamad Sharbatji, Malik Waleed Zeb Khan, Jibran Ikram

**Affiliations:** 1 Department of Medicine, Khyber Medical College, Peshawar, Pakistan; 2 Department of Medicine, Dow University of Health Sciences, Karachi, Pakistan; 3 Department of Internal Medicine, Cooper University Hospital, Camden, NJ USA; 4 Division of Gastroenterology and Hepatology, Department of Medicine, AdventHealth and Loma Linda Regional University, Orlando, USA; 5 Department of Internal Medicine, AdventHealth Hospital and Loma Linda University Regional Campus, Orlando, FL 32804 USA; 6 Department of Medicine, Yale University School of Medicine, New Haven, CT USA; 7 Anesthesiology Department, Outcomes Research Consortium, Cleveland Clinic, Cleveland, OH USA

**Keywords:** Gastric cancer, Malignancy, Cancer mortality, Gastric neoplasm

## Abstract

**Purpose:**

Gastric malignancies remain a significant public health concern and a major contributor to cancer-related mortality worldwide. This study aimed to analyze trends and disparities in gastric malignancy mortality across socio-demographic and regional factors in the United States (US) from 1999 to 2020.

**Methods:**

A retrospective analysis was conducted using CDC WONDER data from 1999 to 2020 for adults aged ≥ 25 years. Data on demographics (age, sex, race/ethnicity), urban–rural classification, and regional trends were extracted. Age-adjusted mortality rates (AAMR) were calculated using the 2000 U.S. population as a reference, with trends analyzed using Joinpoint regression to determine annual percentage changes (APC) with statistical significance (P < 0.05).

**Results:**

Between 1999 and 2020, 276,023 deaths due to gastric malignancies were recorded, with 59.3% occurring among males. The AAMR declined from 7.94 in 1999 to 4.66 in 2020, with an overall AAMR of 5.82. The 65 + age group had the highest AAMR (20.83), while the 25–44 age group had the lowest (0.74). Males consistently reported higher AAMRs (7.60) than females (4.85). NH Black individuals had the highest overall AAMR (10.82), while NH White individuals had the lowest (4.62). Urban areas had higher AAMRs (5.95) than rural areas (5.07).

**Conclusion:**

Mortality from gastric malignancies has declined in the U.S. from 1999 to 2020; however, higher mortality rates in NH Black individuals, males, and urban dwellers highlight the need for targeted interventions and equitable access to prevention and treatment resources. Future research should focus on identifying actionable solutions to mitigate these gaps.

**Supplementary Information:**

The online version contains supplementary material available at 10.1007/s12029-025-01295-9.

## Introduction

Gastric malignancies remain a significant public health concern and a leading cause of cancer-related deaths, with an annual incidence of 9.43 per 100,000 persons in the United States (U.S), according to recent reports [1]. Established risk factors include Helicobacter pylori infections, smoking, obesity, alcohol, high salt intake, coffee use, gastric ulcers, and gastroesophageal reflux disease [2, 3]. Advances in early diagnosis and improved medical management of Helicobacter pylori infection, gastric ulcers, and GERD have resulted in declining mortality rates worldwide [4, 5].

Recent literature shows pronounced disparities in mortality across demographic factors [6, 7]. Males and racial minorities, including Black or African Americans, Asians, and Hispanics, are at higher risk than females and the White population [6, 7]. However, detailed analysis of mortality trends in the U.S is lacking.

We sought to evaluate trends in mortality due to gastric malignancies in the U.S from 1999 to 2020, stratified based on sociodemographic factors including age, sex, race, geographic factors, and urban–rural status.

## Methods

### Study Setting and Population

We conducted a retrospective analysis using the death certificate data from the Centers for Disease Control and Prevention’s Wide-Ranging Online Data for Epidemiologic Research (CDC WONDER) data set [8]. International Statistical Classification of Diseases and Related Health Problems, 10th Revision code (ICD-10), C16, was used to define mortalities due to gastric malignancy in adults (> 25 years). These codes have been well documented in the literature for identifying gastric malignancies [5, 9, 10]. Our study included mortalities among adult individuals in which gastric malignancy was identified as either an underlying or contributory cause of death, encompassing all subtypes classified under ICD-10 code C16. These include malignant neoplasms of the cardia (C16.0), fundus (C16.1), body (C16.2), pyloric antrum (C16.3), and pylorus (C16.4), as well as malignancies involving the lesser curvature (C16.5), greater curvature (C16.6), overlapping sites (C16.8), and unspecified sites of the stomach (C16.9). Ethical approval was not required as the analysis used de-identified publicly available data and adhered to the STROBE (Strengthening the Reporting of Observational Studies in Epidemiology) guidelines for rigorous reporting standards [11].

Sociodemographic data including age, year, race/ethnicity, geographic region, and urban–rural status were extracted. Race/ethnicity included Non-Hispanic (NH) White, NH American Indian/Alaskan Native, NH Black/African American, NH Asian or Pacific Islander, and Hispanic or Latino individuals. Place of death categories included home, hospice, nursing home/long-term care facility, and medical facilities, subdivided into inpatient, outpatient, or emergency room, dead on arrival, and status unknown. The analysis employed the National Center for Health Statistics’ categorization of areas into metropolitan(urban) and non-metropolitan(rural) regions to assess urban–rural trends [12]. For regional comparisons, the U.S. Census Bureau’s designations, Northeast, Midwest, South, and West, were used [12].

### Statistical Analysis

We analyzed mortality related to gastric malignancies from 1999 to 2020 across various sociodemographic and regional variables. Crude mortality rates were calculated as the total number of deaths per 100,000 individuals in the population. Age-adjusted mortality rates (AAMRs) per 100,000 were calculated with the 2000 U.S. population, serving as the standard for AAMR [13]. Trends in AAMRs were assessed using the Joinpoint Regression Program. The software fits a series of connected linear segments on a log-linear scale to identify points where significant changes in mortality trends occur. Model selection was based on the Weighted Bayesian Information Criterion (BIC) method, which balances model complexity and goodness-of-fit and has been shown to perform better than traditional permutation-based methods across a range of situations [14, 15]. For each trend segment, annual percentage changes (APCs) in AAMR were calculated with accompanying 95% confidence intervals (CIs). APCs were interpreted as statistically significant or non-significant based on their statistical deviation from the null hypothesis using a 2-tailed t-test (*P* < 0.05).

## Results

A total of 276,023 deaths associated with gastric malignancies were recorded between 1999 to 2020. 59.3% (163,644) of the deaths occurred among males. NH White (62.3%) was the most common race followed by NH Black (17.2%) (Table 1). The most common subtype of gastric malignancies were unspecified (92.44%), followed by malignancies involving the gastric cardia (7.19%) and the pylorus and pyloric antrum (2.34%).

**Table 1:** Deaths due to Gastric Malignancies in the U.S, stratified by year, sex and race/ethnicity

Year	Overall	Population	Female	Males	American Indian	Asian	Black	White	Hispanic
1999	14,067	180,408,769	5783	8284	69	723	2407	9663	1160
2000	13,929	181,984,640	5871	8058	79	745	2364	9502	1193
2001	13,501	184,305,128	5618	7883	80	724	2248	9142	1270
2002	13,284	186,208,028	5495	7789	96	704	2283	8889	1271
2003	13,205	188,090,429	5506	7699	91	745	2186	8811	1339
2004	12,885	190,205,384	5302	7583	78	729	2170	8447	1437
2005	12,523	192,551,384	5170	7353	92	795	2109	8153	1352
2006	12,330	195,019,359	5118	7212	85	812	2075	7970	1377
2007	12,379	197,403,777	5025	7354	92	805	2154	7915	1405
2008	12,292	199,795,090	4982	7310	71	800	2204	7713	1481
2009	12,031	202,107,016	4847	7184	90	818	2101	7510	1496
2010	12,317	203,891,983	5030	7287	90	820	2139	7597	1647
2011	11,955	206,592,936	4856	7099	82	825	2110	7328	1582
2012	12,087	208,826,037	4924	7163	103	892	2108	7275	1677
2013	12,110	211,085,314	4804	7306	105	854	2069	7321	1738
2014	12,231	213,809,280	4861	7370	113	915	2117	7220	1830
2015	12,160	216,553,817	4881	7279	89	932	2134	7124	1841
2016	12,352	218,641,417	4943	7409	105	1041	2184	7057	1931
2017	12,026	221,447,331	4811	7215	103	1022	2011	6846	2021
2018	12,002	223,311,190	4787	7215	100	915	2152	6759	2050
2019	12,007	224,981,167	4806	7201	109	976	2070	6764	2070
2020	12,350	226,635,013	4959	7391	114	974	2175	6908	2163
Total	**276,023**	**4,473,854,489**	**112,379**	**163,644**	**2036**	**18,566**	**47,570**	**171,914**	**35,331**

### Overall

**Fig. 1:**
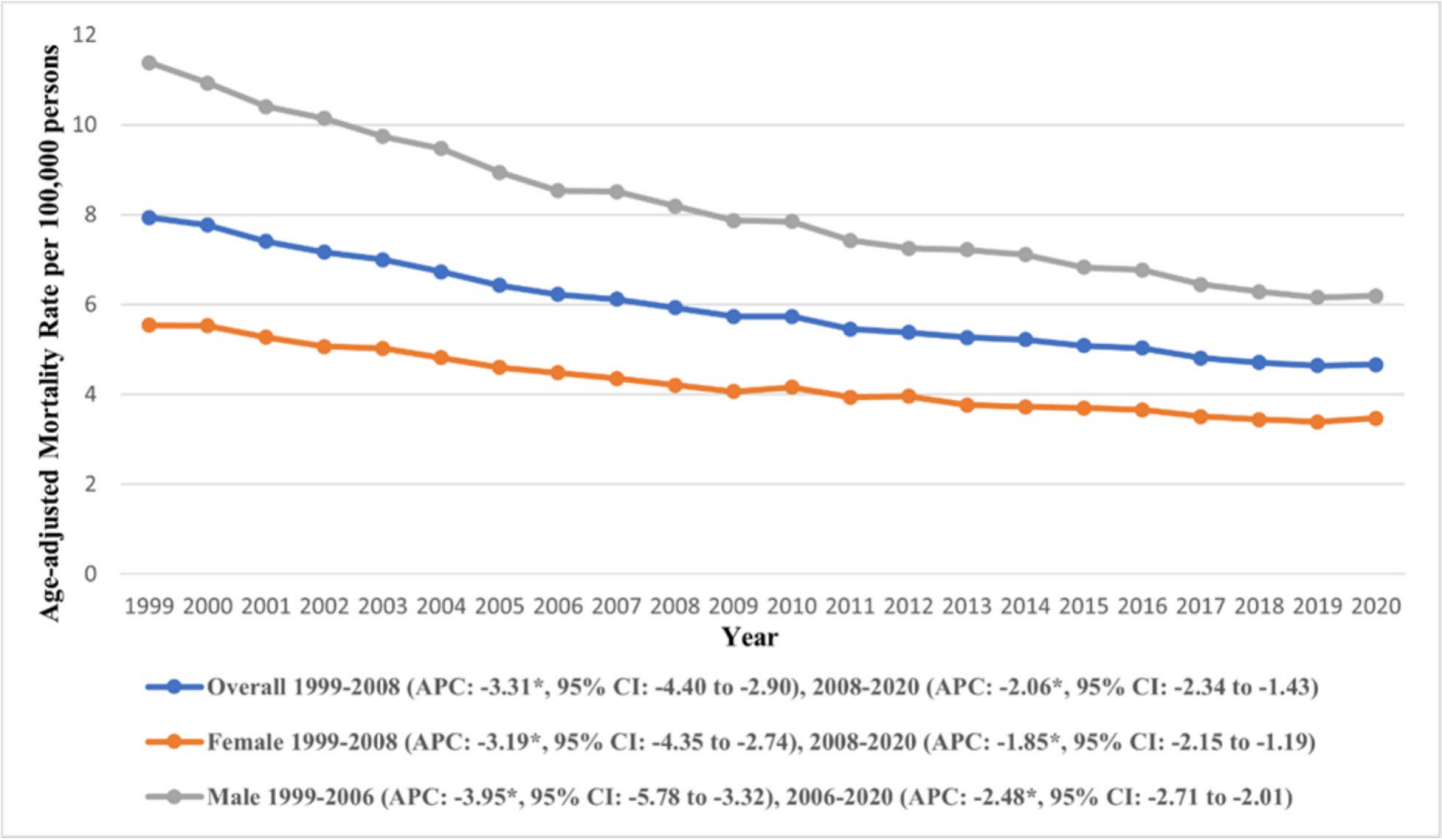
Trends in age-adjusted mortality rate due to gastric malignancies in the U.S. from 1999 to 2020, stratified by year and sex. *Indicates the statistically significant difference of annual percentage change (APC) from 0 at α = 0.05

The overall AAMR for the duration of the study was 5.82 (95% CI: 5.80 to 5.84) (Supplementary Table 1). An overall decline was observed across the study period from 7.94 (95% CI: 7.81 to 8.07) in 1999 to 4.66 (95% CI: 4.58 to 4.75) in 2020 (Fig. 1). The AAMR showed two characteristic trends, in the first period, a steep decline was observed from 1999 to 2008 (APC: −3.31, 95% CI: −4.40 to −2.90), followed by a gentle decline till 2020 (APC: −2.06, 95% CI: −2.34 to −1.43) (Supplementary Table 2).

### Age

**Fig. 2:**
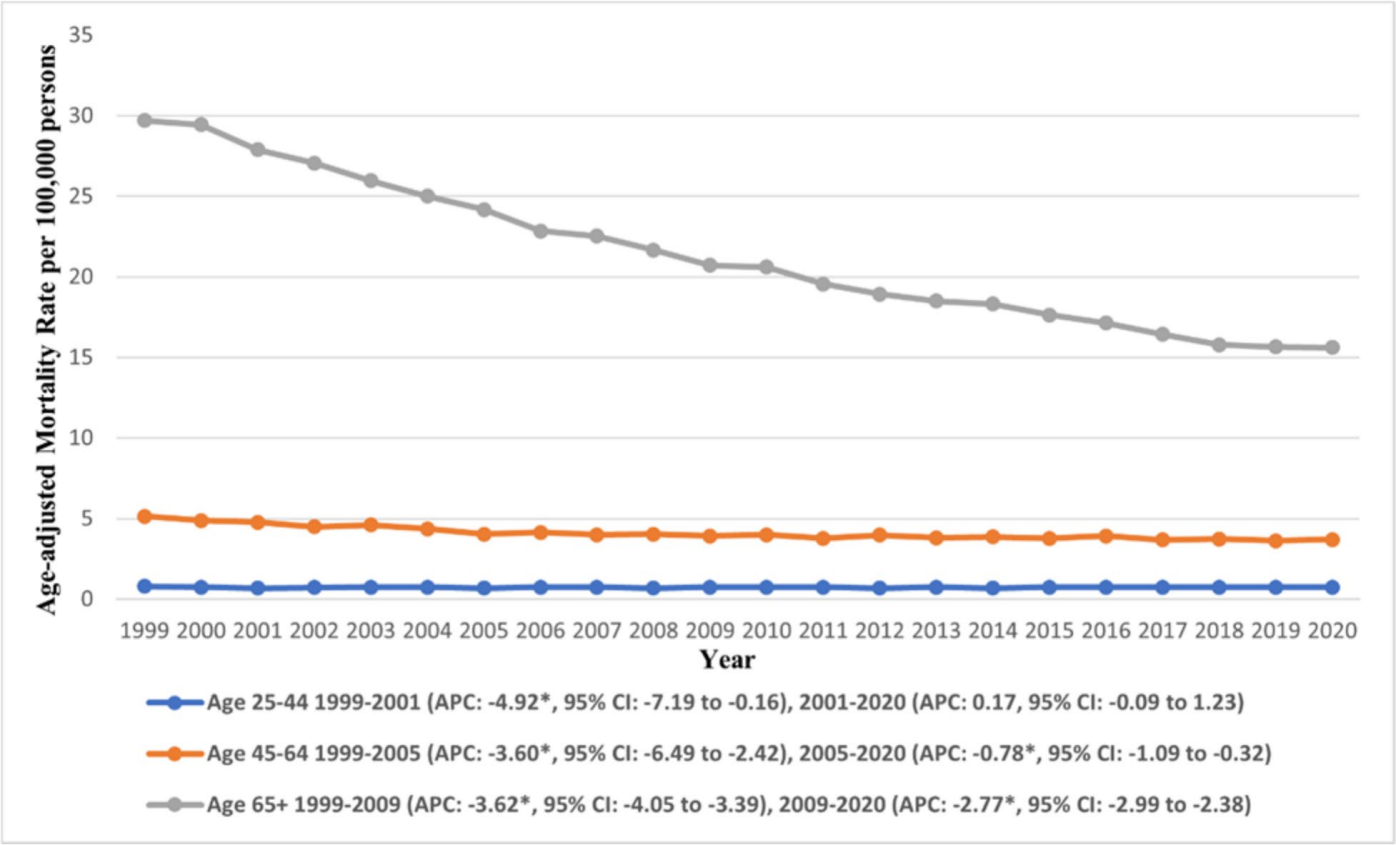
Trends in age-adjusted mortality rate due to gastric malignancies in the U.S. from 1999 to 2020, stratified by age. *Indicates the statistically significant difference of annual percentage change (APC) from 0 at α = 0.05

Our analysis across the 25–44, 45–64, and 65 + age groups revealed marked variations in AAMRs (Fig. 2). The highest AAMR was recorded in the 65 + age group (overall AAMR: 20.83, 95% CI: 20.73 to 20.92) while the lowest was recorded in the 25–44 age group (overall AAMR: 0.74, 95% CI: 0.72 to 0.75) (Supplementary Table 3). Temporal analysis revealed contrasting trends, while the AAMR declined throughout the study period in the 45–64 and 65 + cohorts, the 25–44 age group experienced an initial decline from 1999 to 2001 (APC: −4.92, 95% CI: −7.19 to −0.16) (Supplementary Table 2). Subsequently, the decline reversed and a gentle upward trend prevailed till 2020 (APC: 0.17, 95% CI: −0.09 to 1.23). The largest decline was observed in the 65 + cohort, where the AAMR declined from 29.70 (95% CI: 29.13 to 30.28) in 1999 to 15.63 (95% CI: 15.29 to 15.97) in 2020.

### Gender

Males (overall AAMR: 7.60, 95% CI: 7.56 to 7.63) consistently reported higher AAMRs compared to females (overall AAMR: 4.85, 95% CI: 4.82 to 4.87) **(**Fig. 1**)**. A similar trend was observed in both groups, with an initially steep decline till 2006/08 (Male APC 1999–2006: −3.95, 95% CI: −5.78 to −3.32, Female APC 1999–2008: −3.19, 95% CI: −4.34 to −2.74), followed by a gentle decline in the latter part of the study period (Male APC 2006–2020: −2.48, 95% CI: −2.71 to −2.01, Female APC 2008–2020: −1.85, 95% CI: −2.15 to −1.19) (Supplementary Tables 1 and 2). A more significant decline was observed among males compared to females.

### Race

Profound variations were observed across the racial groups. NH Black (overall AAMR: 10.82, 95% CI: 10.72 to 10.92) reported the highest AAMR, followed by NH Asian or Pacific Islander (overall AAMR: 9.94, 95% CI: 9.79 to 10.08), Hispanic or Latino (overall AAMR: 9.03, 95% CI: 8.93 to 9.13) and NH American Indian or Alaska Natives (overall AAMR: 7.67, 95% CI: 7.32 to 8.02) (Supplementary Table 4). The lowest AAMR was reported amongst the NH White cohort (overall AAMR: 4.62, 95% CI: 4.60 to 4.64).

**Fig. 3:**
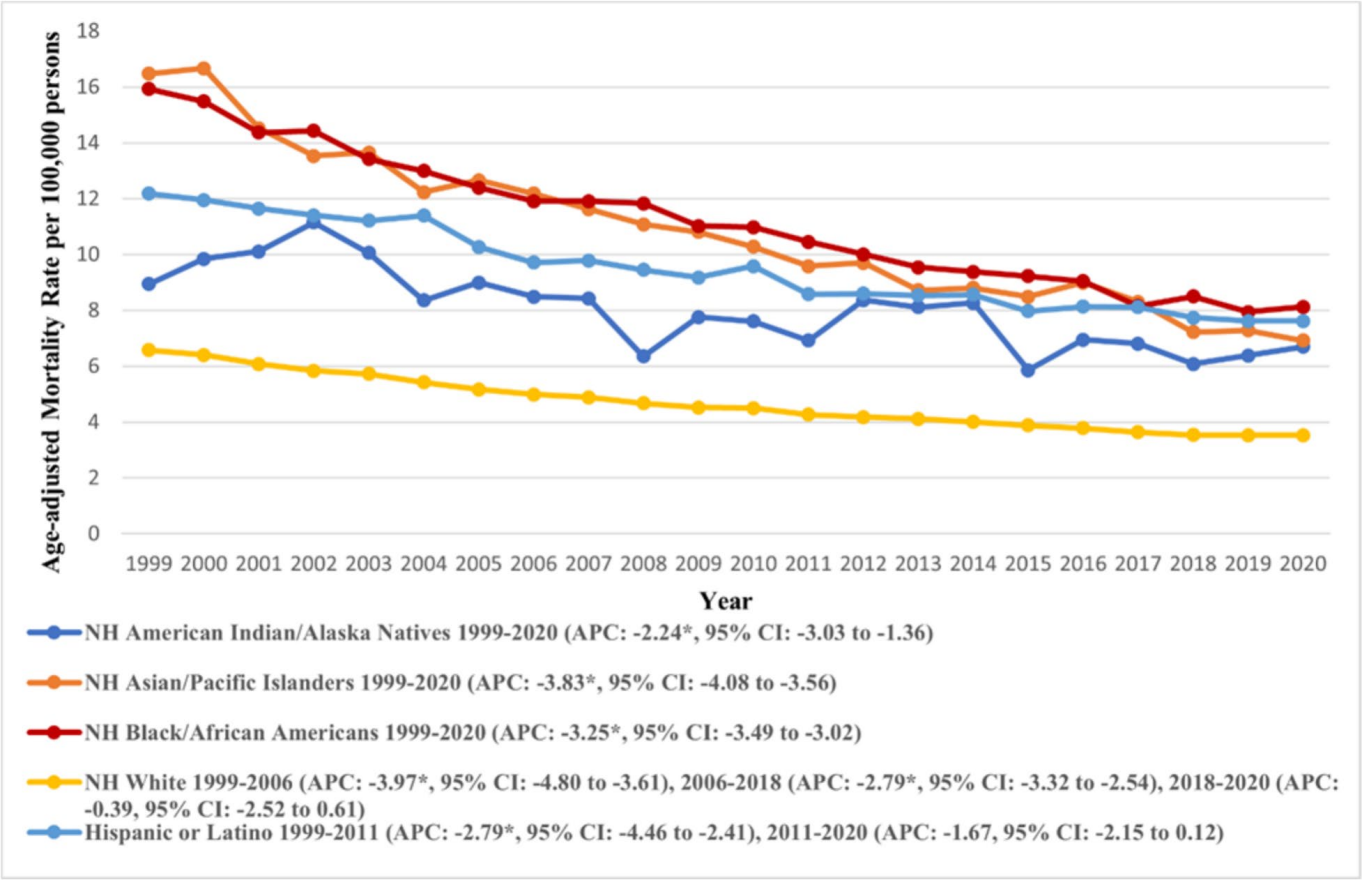
Trends in age-adjusted mortality rate due to gastric malignancies in the U.S. from 1999 to 2020, stratified by race. *Indicates the statistically significant difference of annual percentage change (APC) from 0 at α = 0.05

All racial groups observed a decline in their AAMRs, with the largest decline observed among NH Asians, whose AAMRs declined from 16.47 (95% CI: 15.20 to 17.75) in 1999 to 6.92 (95% CI: 6.48 to 7.36) in 2020 (APC: −3.83, 95% CI: −4.08 to −3.56) (Supplementary Table 2). Similar trends were observed across other groups. The trends in AAMR across each racial group are depicted in Fig. 3.

### State

AAMRs varied across states with Hawaii (overall AAMR: 9.65, 95% CI: 9.25 to 10.06) reporting the highest, followed by District of Columbia (overall AAMR: 8.72, 95% CI: 8.09 to 9.35). The lowest, on the other hand were recorded in Wyoming (overall AAMR: 3.66, 95% CI: 3.24 to 4.07).

**Fig. 4:**
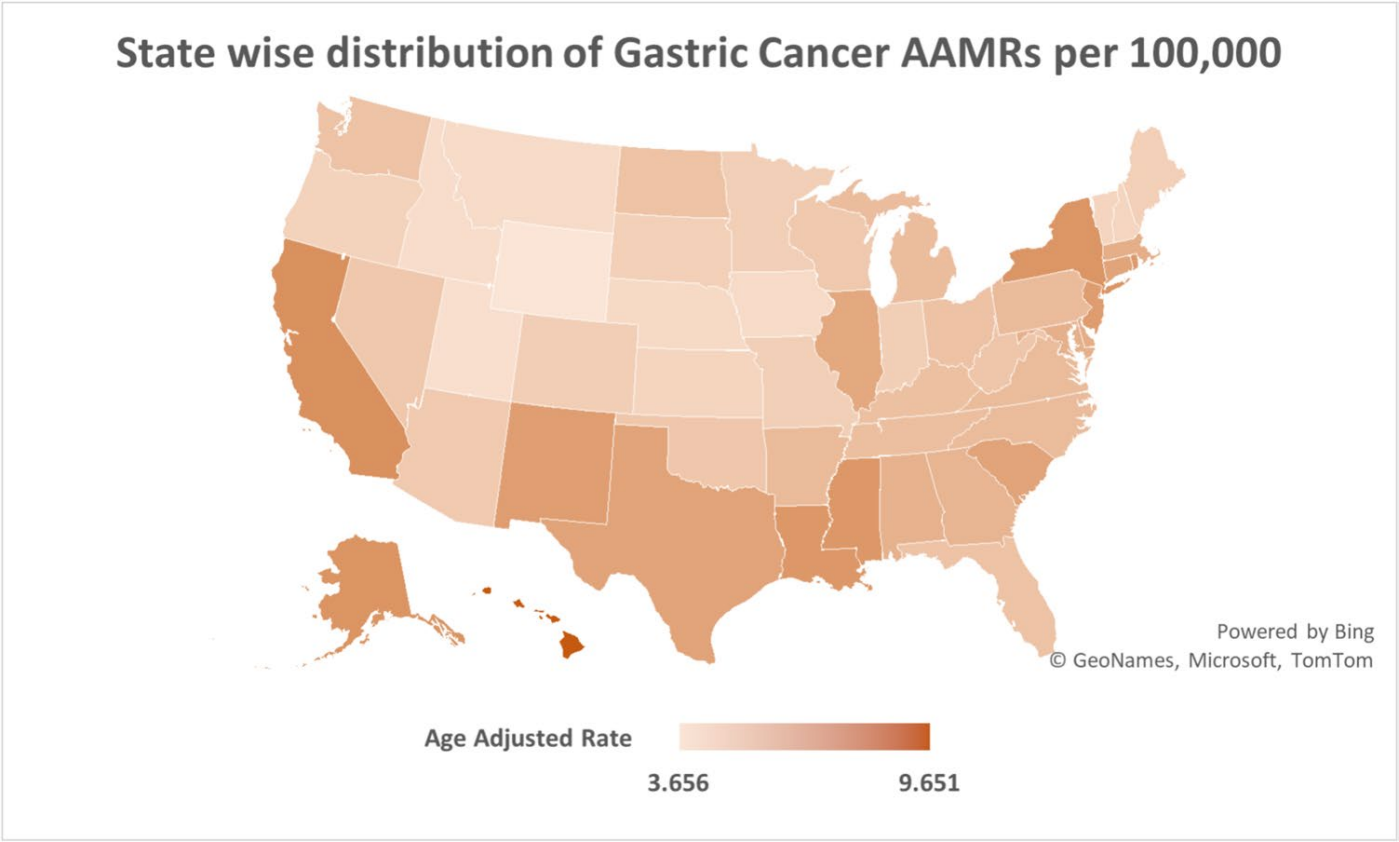
Map of the Unites states depicting state-wise variations in age-adjusted mortality rates due to gastric malignancies in the United States

All states recorded a decline in AAMRs, however the declining trend either plateaued or reversed towards the end of the study period. In California, a steep decline took place from 1999 to 2004 (APC: −3.46, 95% CI: −6.06 to −2.07), followed by a gentle decline till 2018 (APC: −1.93, 95% CI: −3.59 to −1.01). The subsequent period saw a reversal in decline, with the AAMRs increasing till 2020 (APC: 2.02, 95% CI: −1.77 to 4.06). A map of the U.S depicting variations in AAMR across states is presented in Fig. 4.

### Census Regions

**Fig. 5:**
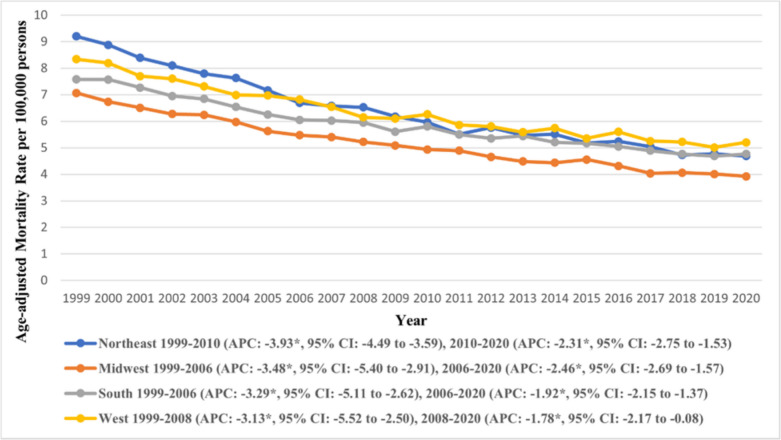
Trends in age-adjusted mortality rate due to gastric malignancies in the U.S. from 1999 to 2020, stratified by census regions. *Indicates the statistically significant difference of annual percentage change (APC) from 0 at α = 0.05

Across the four U.S Census Regions, the Northeast reported the highest AAMRs (overall AAMR: 6.29, 95% CI: 6.23 to 6.34), followed by the West (overall AAMR: 6.19, 95% CI: 6.14 to 6.23) and the South (overall AAMR: 5.72, 95% CI: 5.69 to 5.76), whereas the lowest were recorded in the Midwest (overall AAMR: 5.08, 95% CI: 5.04 to 5.12) **(**Supplementary Table 5). A declining trend was observed across the regions, with the decline becoming gentler towards the end of the study period. In the West, the AAMR declined steeply from 1999 to 2008 (APC: −3.13, 95% CI: −5.52 to −2.50), the subsequent period saw a decline in the downward trend (APC: −1.78, 95% CI: −2.17 to −0.08) (Supplementary Table 2). Trends in AAMR stratified by urban–rural status are depicted in Fig. 5.

### Urbanization

**Fig. 6:**
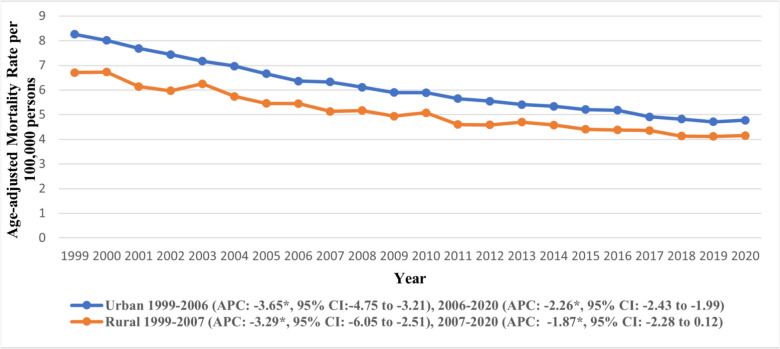
Trends in age-adjusted mortality rate due to gastric malignancies in the U.S. from 1999 to 2020, stratified by urban-rural status. *Indicates the statistically significant difference of annual percentage change (APC) from 0 at α = 0.05

Our analysis across urban–rural status revealed notable variations. Urban areas had the higher AAMRs (overall AAMR: 5.95, 95% CI: 5.92 to 5.97) than rural areas, including (overall AAMR: 5.07, 95% CI: 5.02 to 5.12) (Supplementary Table 6). A declining trend was observed across all areas, which plateaued towards the end of the study period. In rural areas the AAMR declined steeply from 1999 to 2007 (APC: −3.29, 95% CI: −6.05 to −2.51), after which a gentle decline persisted till 2020 (APC: −1.87, 95% CI: −2.28 to 0.12) (Supplementary Table 2). Trends in AAMR stratified by urban–rural status are depicted in Fig. 6.

## Discussion

In this 20-year analysis of mortality data from the Centers for Disease Control and Prevention in the United States, we report key findings concerning gastric malignancy-related mortality in adults aged 25 and above from 1999 to 2020. First, there was an overall decline in mortality rates from 1999–2020. Mortality rates varied significantly across age groups, with the highest AAMR observed in individuals aged 65 +, while the lowest was recorded in the 25–44 age group. Second, males consistently exhibited higher mortality rates throughout the study duration as compared to females. Third, NH Black or African American individuals reported the highest mortality rates relative to other racial groups. Fourth, significant regional and geographical disparities were observed, with the highest AAMRs in Hawaii and the Northeast region. Finally, the analysis demonstrated higher gastric malignancy incidence and mortality rates in urban areas as compared to a lower rate in rural areas throughout the study period. These findings hold significant implications for public health policy.

The study revealed a substantial reduction in mortality rates between 1999 and 2020, consistent with findings from other studies on gastrointestinal cancer trends [16, 17]. This decline is multifactorial and can be attributed to advancements in chemotherapeutic regimens incorporating fluoropyrimidine, platinum agents, and taxanes, which have successfully improved the 5-year survival of gastric cancer in the United States to 31% [18]. Moreover, improved treatments and early detection methods may contribute to declining mortality [19, 20]. For instance, the widespread eradication treatments for Helicobacter Pylori, a well-established risk factor for gastric cancer, have played an essential role in lowering incidence and associated mortality [21]. The adoption of minimally invasive surgery and endoscopic treatment techniques [22] and novel anti-HER2 therapeutic drugs, such as T-DXd and disitamab vedotin (RC48), has further led to significant advances in the treatment of gastric cancer [23]. These advancements have significantly improved patient outcomes over the last two decades.

Age emerged as a pivotal determinant in gastric malignancy-related mortality, with the highest mortality rates observed in individuals aged 65 and older. About 6 of every 10 people diagnosed with stomach cancer each year are 65 or older [24]. Older people tend to experience poorer survival outcomes due to their reduced ability to tolerate the complications of metastatic diseases, influenced by baseline advanced comorbidities. This is further exacerbated by physicians being less aggressive in the treatment of older patients, whether surgical or chemotherapy [25]. Geriatric patients tend to experience a higher rate of postoperative complications compared to younger individuals, making them less ideal candidates for surgical intervention [26]. Additionally, aging is associated with immunosenescence, which is linked to both decreased ability to suppress malignant cell growth and increased mortality [27].

Our findings reveal a persistent and significant gender disparity in gastric cancer mortality, with males exhibiting higher AAMRs than females throughout the study period. Globally, in 2020, the mortality rate for stomach cancer was 11.0 per 100,000 in males, compared to 4.9 per 100,000 in females [28]. Several behavioral and biological factors may drive this disparity; compared to females, males tend to have a greater prevalence of risk factors such as tobacco and alcohol consumption, both of which have been associated with increased gastric cancer incidence and mortality [29]. A meta-analysis of 42 studies estimated that smokers face an approximately 1.53-fold higher risk of developing gastric cancer, with this risk being more pronounced in males than females [30]. In contrast, the lower AAMRs exhibited by females may be attributed to the protective role of estrogen in reducing the risk of gastric cancer by inhibiting gastric cancer cell proliferation and inflammatory response modulation [31, 32]. These hormonal alterations may influence disease development and overall survival rates among genders, signifying the need for tailored interventions for males to address the higher mortality.

NH Black or African American individuals had higher rates of mortality in comparison to other racial and ethnic groups, a disparity influenced by biological, socioeconomic, and healthcare access factors. Previous studies have demonstrated that Black patients display a higher incidence of gastric cancer and associated mortality [33]. One key contributor to this disparity is the persistently high prevalence of H. pylori infection in Blacks, a well-established risk factor for gastric malignancy [34]. Additionally, the switch from legume and grain diets to Westernized high-caloric diets has been linked to increased cancer risk, particularly in genetically susceptible populations [35]. Inequities in healthcare access compound these predispositions; according to Bakkila et al.[36], even when Black patients overcome challenges to diagnosis and treatment and accept adjunct medicines such as chemotherapy, they may still receive inadequate care. Addressing these discrepancies necessitates a multifaceted approach that includes increased healthcare access and culturally appropriate interventions.

Our results also showed specific regional and geographical disparities. The highest AAMRs were observed in the state of Hawaii and the Northeast region. These findings align with previous studies reporting high incidence in these areas, which can be explained by H. pylori infection, cigarette smoking, and lower levels of pepsinogen and ferritin in these populations [37, 38]. Additionally, our findings displayed a higher mortality rate in metropolitan areas. Previous studies have reported a higher incidence of gastric cancer in urban areas [39, 40]. However, rural areas had worse survival outcomes due to the lack of proper diagnostic and screening systems, education, and access to healthcare oncological services [39, 40]. The elevated mortality in metropolitan areas can be explained by the higher rates of dietary risk factors in urban populations, including consumption of processed and high-salt food, and lack of diets rich in fiber and antioxidants [41, 42]. These findings underscore the need for lifestyle modifications to tackle the challenges faced by the urban population in reducing gastric cancer mortality.

Addressing gastric malignancy-related mortality, especially in high-risk individuals, remains a critical issue. Multidisciplinary European and UK endoscopy recommendations indicate that individuals with a family history of stomach cancer or chronic H. pylori-associated gastritis should undergo endoscopic surveillance with guided biopsies every three years [43]. Endoscopic biopsies, the gold standard for diagnosis, should be carried out to provide adequate information for histological and molecular interpretation [44]. Improving dietary habits, incorporating fruits and vegetables, and eradicating H. pylori are a few preventive measures. The International Agency for Research on Cancer also suggests an increased consumption of fruits and vegetables to lower the risk of gastric cancer [45]. Tackling racial and ethnic disparities remains prudent for equitable access to healthcare and healthy food. Age-adapted treatment approaches are required since the older population faces the highest mortality.

### Limitations

This study has several limitations, that must be considered. Relying on ICD-10 codes to identify gastric malignancies as a cause of death introduces a possibility of misclassification bias due to coding errors. The database lacks data regarding clinical and laboratory measures that can accurately assess the severity of the illness. Furthermore, there is a shortage of knowledge of medical treatments and remedies, with no data on co-morbidities, insurance, or patient medication. The absence of data on healthcare access, particularly in rural and underserved areas, limits our understanding of geographic inequalities in gastric cancer mortality. Finally, socioeconomic determinants of health, which may impact access to care, are not adequately recorded.

## Conclusion

In conclusion, there was a significant decrease in the overall age-adjusted mortality rate linked to gastric cancer between 1999 and 2020. It is noteworthy that males, Black/African Americans, adults aged 65 +, and those living in the Northeast and metropolitan areas of the US had the highest AAMRs. Targeted interventions are necessary to address and reduce the rates of gastric malignancy-related mortality among adults. Future research should focus on developing individualized risk stratification models that incorporate genetic predispositions, comorbidities, healthcare inequities, and specific treatment regimens to better predict and reduce gastric cancer risk specifically in the elderly, male, and Black populations.

## Supplementary Information

Below is the link to the electronic supplementary material.

ESM 1 DOCX (152 KB)

## Data Availability

Data is provided within the manuscript and supplementary information files.
