# Supplementary material for: Trends and Disparities in Mortality Due to Gastric Malignancies the United States: A Nationwide Analysis from 1999 to 2020
Source: J Gastrointest Cancer. 2025 Aug 5;56(1):168. doi: 10.1007/s12029-025-01295-9 (PMC12325418; doi:10.1007/s12029-025-01295-9)
Supplement: Supplementary file 1 — DOCX (152 KB) [file 12029_2025_1295_MOESM1_ESM.docx]

**Supplementary File**

**Trends and Disparities in Mortality due to Gastric Malignancies the United States: A Nationwide Analysis from 1999 to 2020**

**Authors:**

**Muhammad Ahmad^1^, Aizaz Ali^1^, Tahreem Mari^2^, Fariha Hasan^3^, Saeed ali^4^, Mohamad Sharbatji^5^, Malik Waleed Zeb Khan^6^, Jibran Ikram^7^**

**Affiliations**

**^1^Department of Medicine, Khyber Medical College, Peshawar, Pakistan**

**^2^Department of Medicine, Dow University of Health Sciences, Karachi, Pakistan**

**^3^Department of Internal Medicine, Cooper University Hospital, NJ, USA**

**^4^Department of Internal Medicine, AdventHealth Hospital and Loma Linda University Regional Campus, Orlando, FL, 32804, USA**

**^5^Division of Gastroenterology and Hepatology, Department of Medicine, University of Illinois at Chicago, Chicago, IL, United States**

**^6^Department of Medicine, Yale University School of Medicine, New Haven, USA**

**^7^Outcomes Research Consortium, Anesthesiology Department, Cleveland Clinic, Cleveland, Ohio, USA**

**Corresponding author:**

**Jibran Ikram**

**Outcomes Research Consortium, Anesthesiology Department, Cleveland Clinic, Cleveland, Ohio, USA**

[**IKRAMJ@ccf.org**](mailto:IKRAMJ@ccf.org)

| **Year** | **Age Adjusted Mortality Rate (95% CI)** | | |
| --- | --- | --- | --- |
|  | **Overall** | **Male** | **Female** |
| 1999 | 7.94 (7.81 to 8.07) | 11.39 (11.14 to 11.64) | 5.54 (5.40 to 5.69) |
| 2000 | 7.77 (7.64 to 7.90) | 10.93 (10.69 to 11.17) | 5.53 (5.39 to 5.67) |
| 2001 | 7.41 (7.28 to 7.53) | 10.41 (10.17 to 10.64) | 5.27 (5.13 to 5.41) |
| 2002 | 7.17 (7.05 to 7.29) | 10.15 (9.92 to 10.37) | 5.07 (4.93 to 5.20) |
| 2003 | 7.00 (6.88 to 7.12) | 9.74 (9.52 to 9.96) | 5.03 (4.89 to 5.16) |
| 2004 | 6.73 (6.61 to 6.85) | 9.47 (9.25 to 9.69) | 4.82 (4.69 to 4.95) |
| 2005 | 6.43 (6.31 to 6.54) | 8.94 (8.73 to 9.15) | 4.61 (4.48 to 4.73) |
| 2006 | 6.22 (6.11 to 6.33) | 8.53 (8.33 to 8.73) | 4.48 (4.36 to 4.61) |
| 2007 | 6.12 (6.01 to 6.23) | 8.52 (8.32 to 8.71) | 4.36 (4.23 to 4.48) |
| 2008 | 5.93 (5.83 to 6.04) | 8.19 (8.00 to 8.38) | 4.21 (4.09 to 4.33) |
| 2009 | 5.74 (5.63 to 5.84) | 7.87 (7.69 to 8.06) | 4.07 (3.95 to 4.18) |
| 2010 | 5.74 (5.64 to 5.84) | 7.85 (7.67 to 8.04) | 4.16 (4.04 to 4.28) |
| 2011 | 5.46 (5.36 to 5.56) | 7.43 (7.25 to 7.60) | 3.94 (3.82 to 4.05) |
| 2012 | 5.38 (5.28 to 5.48) | 7.26 (7.08 to 7.43) | 3.96 (3.85 to 4.07) |
| 2013 | 5.27 (5.17 to 5.36) | 7.22 (7.05 to 7.39) | 3.76 (3.66 to 3.87) |
| 2014 | 5.22 (5.13 to 5.31) | 7.11 (6.95 to 7.28) | 3.73 (3.62 to 3.83) |
| 2015 | 5.09 (4.99 to 5.18) | 6.83 (6.67 to 6.99) | 3.70 (3.59 to 3.80) |
| 2016 | 5.03 (4.94 to 5.13) | 6.77 (6.61 to 6.93) | 3.66 (3.55 to 3.76) |
| 2017 | 4.81 (4.73 to 4.90) | 6.45 (6.29 to 6.60) | 3.51 (3.41 to 3.61) |
| 2018 | 4.71 (4.63 to 4.80) | 6.29 (6.14 to 6.43) | 3.44 (3.34 to 3.54) |
| 2019 | 4.64 (4.56 to 4.73) | 6.16 (6.01 to 6.30) | 3.39 (3.29 to 3.49) |
| 2020 | 4.66 (4.58 to 4.75) | 6.20 (6.05 to 6.34) | 3.47 (3.37 to 3.57) |

**Supplementary Table 1. Age-adjusted mortality rate due to Gastrointestinal cancer in the U.S stratified by year and sex.**

| **Subgroup** | **Year** | **APC (95% CI)** |
| --- | --- | --- |
| **Overall** | 1999–2008 | -3.31 (-4.40 to -2.90) |
|  | 2008–2020 | -2.06 (-2.34 to -1.43) |
| **Age 25–44 years** | 1999–2001 | -4.92 (-7.19 to -0.16) |
|  | 2001–2020 | 0.17 (-0.09 to 1.23) |
| **Age 45–64 years** | 1999–2005 | -3.60 (-6.49 to -2.42) |
|  | 2005–2020 | -0.78 (-1.09 to -0.32) |
| **Age >65 years** | 1999–2009 | -3.62 (-4.05 to -3.39) |
|  | 2009–2020 | -2.77 (-2.99 to -2.38) |
| **Male** | 1999–2006 | -3.95 (-5.78 to -3.32) |
|  | 2006–2020 | -2.48 (-2.71 to -2.01) |
| **Female** | 1999–2008 | -3.19 (-4.35 to -2.74) |
|  | 2008–2020 | -1.85 (-2.15 to -1.19) |
| **NH American Indian/Alaska Native** | 1999–2020 | -2.24 (-3.03 to -1.36) |
| **NH Asian/Pacific Islander** | 1999–2020 | -3.83 (-4.08 to -3.56) |
| **NH Black/African American** | 1999–2020 | -3.25 (-3.49 to -3.02) |
| **NH White** | 1999–2006 | -3.97 (-4.80 to -3.61) |
|  | 2006–2018 | -2.79 (-3.32 to -2.54) |
|  | 2018–2020 | -0.39 (-2.52 to 0.61) |
| **Hispanic or Latino** | 1999–2011 | -2.79 (-4.46 to -2.41) |
|  | 2011–2020 | -1.67 (-2.15 to 0.12) |
| **Northeast Region** | 1999–2010 | -3.93 (-4.49 to -3.59) |
|  | 2010–2020 | -2.31 (-2.75 to -1.53) |
| **Midwest Region** | 1999–2006 | -3.48 (-5.40 to -2.91) |
|  | 2006–2020 | -2.46 (-2.69 to -1.57) |
| **South Region** | 1999–2006 | -3.29 (-5.11 to -2.62) |
|  | 2006–2020 | -1.92 (-2.15 to -1.37) |
| **West Region** | 1999–2008 | -3.13 (-5.52 to -2.50) |
|  | 2008–2020 | -1.78 (-2.17 to -0.08) |
| **Non-metropolitan (rural)** | 1999-2007 | -3.29 (-6.05 to -2.51) |
|  | 2007-2020 | -1.87 (-2.28 to 0.12) |
| **Metropolitan (urban)** | 1999-2006 | -3.65 (-4.75 to -3.21) |
|  | 2006-2020 | -2.26 (-2.43 to -1.99) |

**Supplementary Table 2. Annual Percentage Changes (95% CI) in AAMR stratified by age, sex, race/ethnicity, census region and urban-rural status in the U.S from 1999-2020**

| **Year** | **Age Adjusted Mortality Rate (95% CI)** | | |
| --- | --- | --- | --- |
|  | **>65** | **45-64** | **25-44** |
| 1999 | 29.70 (29.13–30.28) | 5.14 (4.96–5.32) | 0.79 (0.73–0.85) |
| 2000 | 29.44 (28.87–30.01) | 4.89 (4.71–5.06) | 0.74 (0.68–0.79) |
| 2001 | 27.90 (27.35–28.45) | 4.77 (4.60–4.94) | 0.68 (0.63–0.74) |
| 2002 | 27.07 (26.53–27.61) | 4.49 (4.33–4.65) | 0.73 (0.67–0.79) |
| 2003 | 25.96 (25.44–26.49) | 4.61 (4.45–4.77) | 0.74 (0.68–0.80) |
| 2004 | 25.01 (24.50–25.52) | 4.37 (4.22–4.52) | 0.74 (0.68–0.80) |
| 2005 | 24.17 (23.67–24.68) | 4.04 (3.89–4.18) | 0.68 (0.63–0.74) |
| 2006 | 22.84 (22.36–23.32) | 4.14 (3.99–4.28) | 0.74 (0.68–0.80) |
| 2007 | 22.54 (22.06–23.01) | 4.00 (3.86–4.14) | 0.74 (0.68–0.80) |
| 2008 | 21.66 (21.20–22.13) | 4.04 (3.90–4.17) | 0.68 (0.63–0.74) |
| 2009 | 20.72 (20.27–21.17) | 3.92 (3.78–4.05) | 0.74 (0.68–0.80) |
| 2010 | 20.61 (20.16–21.05) | 4.00 (3.86–4.13) | 0.74 (0.68–0.80) |
| 2011 | 19.55 (19.12–19.98) | 3.78 (3.65–3.91) | 0.74 (0.68–0.80) |
| 2012 | 18.93 (18.52–19.35) | 3.98 (3.85–4.11) | 0.68 (0.62–0.74) |
| 2013 | 18.51 (18.11–18.92) | 3.82 (3.69–3.95) | 0.74 (0.68–0.80) |
| 2014 | 18.31 (17.92–18.71) | 3.86 (3.73–3.99) | 0.68 (0.62–0.74) |
| 2015 | 17.64 (17.26–18.03) | 3.78 (3.65–3.91) | 0.74 (0.68–0.80) |
| 2016 | 17.14 (16.76–17.51) | 3.92 (3.79–4.05) | 0.74 (0.68–0.80) |
| 2017 | 16.43 (16.07–16.79) | 3.68 (3.55–3.81) | 0.74 (0.68–0.80) |
| 2018 | 15.79 (15.44–16.14) | 3.74 (3.61–3.87) | 0.74 (0.68–0.80) |
| 2019 | 15.65 (15.31–15.99) | 3.62 (3.49–3.75) | 0.74 (0.68–0.80) |
| 2020 | 15.63 (15.29–15.97) | 3.70 (3.57–3.83) | 0.74 (0.68–0.80) |

**Supplementary Table 3. Age-adjusted mortality rate due to Gastrointestinal cancer in the U.S stratified by age.**

| **Year** | **Age Adjusted Mortality Rate (95% CI)** | | | | |
| --- | --- | --- | --- | --- | --- |
|  | **American Indian or Alaska Native** | **Asian or Pacific Islander** | **Black or African American** | **White** | **Hispanic or Latino** |
| 1999 | 8.94 [6.86 to 11.46] | 16.47 [15.20 to 17.75] | 15.93 [15.29 to 16.58] | 6.58 [6.44 to 6.71] | 12.19 [11.44 to 12.93] |
| 2000 | 9.84 [7.70 to 12.39] | 16.66 [15.41 to 17.92] | 15.48 [14.85 to 16.11] | 6.41 [6.28 to 6.54] | 11.95 [11.23 to 12.67] |
| 2001 | 10.11 [7.91 to 12.73] | 14.52 [13.41 to 15.63] | 14.37 [13.76 to 14.97] | 6.08 [5.96 to 6.21] | 11.64 [10.96 to 12.32] |
| 2002 | 11.15 [8.89 to 13.81] | 13.53 [12.49 to 14.58] | 14.44 [13.83 to 15.04] | 5.84 [5.72 to 5.96] | 11.41 [10.74 to 12.08] |
| 2003 | 10.06 [7.96 to 12.53] | 13.65 [12.63 to 14.68] | 13.42 [12.84 to 13.99] | 5.73 [5.61 to 5.85] | 11.21 [10.57 to 11.85] |
| 2004 | 8.36 [6.48 to 10.61] | 12.24 [11.31 to 13.16] | 13.00 [12.44 to 13.55] | 5.42 [5.31 to 5.54] | 11.39 [10.77 to 12.02] |
| 2005 | 9.00 [7.13 to 11.20] | 12.66 [11.75 to 13.58] | 12.40 [11.86 to 12.94] | 5.17 [5.06 to 5.28] | 10.27 [9.69 to 10.85] |
| 2006 | 8.48 [6.66 to 10.65] | 12.18 [11.31 to 13.05] | 11.92 [11.39 to 12.44] | 4.99 [4.88 to 5.10] | 9.72 [9.17 to 10.26] |
| 2007 | 8.43 [6.69 to 10.50] | 11.63 [10.80 to 12.46] | 11.91 [11.39 to 12.43] | 4.89 [4.78 to 5.00] | 9.79 [9.25 to 10.33] |
| 2008 | 6.35 [4.87 to 8.15] | 11.08 [10.29 to 11.88] | 11.83 [11.32 to 12.34] | 4.67 [4.57 to 4.78] | 9.45 [8.94 to 9.97] |
| 2009 | 7.77 [6.14 to 9.69] | 10.80 [10.04 to 11.57] | 11.02 [10.53 to 11.50] | 4.52 [4.42 to 4.63] | 9.17 [8.68 to 9.67] |
| 2010 | 7.61 [6.03 to 9.49] | 10.29 [9.56 to 11.01] | 10.98 [10.50 to 11.46] | 4.50 [4.40 to 4.61] | 9.59 [9.10 to 10.08] |
| 2011 | 6.92 [5.43 to 8.71] | 9.59 [8.91 to 10.26] | 10.45 [9.99 to 10.91] | 4.27 [4.17 to 4.36] | 8.58 [8.13 to 9.03] |
| 2012 | 8.37 [6.66 to 10.09] | 9.71 [9.05 to 10.36] | 10.01 [9.57 to 10.45] | 4.18 [4.08 to 4.28] | 8.61 [8.17 to 9.04] |
| 2013 | 8.11 [6.48 to 9.75] | 8.73 [8.13 to 9.33] | 9.54 [9.12 to 9.97] | 4.11 [4.02 to 4.21] | 8.54 [8.12 to 8.96] |
| 2014 | 8.26 [6.66 to 9.87] | 8.80 [8.22 to 9.39] | 9.38 [8.97 to 9.80] | 4.01 [3.91 to 4.10] | 8.56 [8.15 to 8.98] |
| 2015 | 5.86 [4.65 to 7.29] | 8.49 [7.94 to 9.05] | 9.23 [8.83 to 9.63] | 3.89 [3.79 to 3.98] | 7.97 [7.59 to 8.35] |
| 2016 | 6.95 [5.57 to 8.33] | 8.99 [8.44 to 9.55] | 9.05 [8.66 to 9.44] | 3.78 [3.69 to 3.87] | 8.14 [7.76 to 8.52] |
| 2017 | 6.81 [5.44 to 8.18] | 8.31 [7.79 to 8.83] | 8.16 [7.79 to 8.53] | 3.65 [3.56 to 3.74] | 8.11 [7.74 to 8.48] |
| 2018 | 6.08 [4.85 to 7.31] | 7.22 [6.75 to 7.70] | 8.50 [8.13 to 8.87] | 3.54 [3.46 to 3.63] | 7.74 [7.39 to 8.09] |
| 2019 | 6.38 [5.15 to 7.62] | 7.28 [6.82 to 7.75] | 7.94 [7.59 to 8.29] | 3.53 [3.45 to 3.62] | 7.62 [7.28 to 7.96] |
| 2020 | 6.69 [5.43 to 7.96] | 6.92 [6.48 to 7.36] | 8.13 [7.77 to 8.48] | 3.53 [3.44 to 3.62] | 7.62 [7.29 to 7.96] |

**Supplementary Table 4. Age-adjusted mortality rate due to Gastrointestinal cancer in the U.S stratified by race.**

| **Year** | **Age Adjusted Mortality Rate (95% CI)** | | | |
| --- | --- | --- | --- | --- |
|  | **Northeast** | **Midwest** | **South** | **West** |
| 1999 | 9.20 [8.89 to 9.51] | 7.06 [6.81 to 7.32] | 7.58 [7.36 to 7.79] | 8.34 [8.04 to 8.64] |
| 2000 | 8.88 [8.58 to 9.18] | 6.74 [6.49 to 6.99] | 7.57 [7.36 to 7.79] | 8.20 [7.90 to 8.49] |
| 2001 | 8.39 [8.10 to 8.69] | 6.51 [6.27 to 6.75] | 7.27 [7.06 to 7.47] | 7.70 [7.41 to 7.98] |
| 2002 | 8.10 [7.81 to 8.38] | 6.27 [6.04 to 6.51] | 6.95 [6.75 to 7.15] | 7.61 [7.33 to 7.88] |
| 2003 | 7.80 [7.52 to 8.08] | 6.24 [6.01 to 6.47] | 6.84 [6.64 to 7.04] | 7.31 [7.04 to 7.58] |
| 2004 | 7.63 [7.35 to 7.90] | 5.98 [5.75 to 6.20] | 6.54 [6.35 to 6.73] | 6.99 [6.73 to 7.25] |
| 2005 | 7.17 [6.90 to 7.43] | 5.63 [5.41 to 5.85] | 6.25 [6.07 to 6.44] | 6.97 [6.71 to 7.23] |
| 2006 | 6.70 [6.44 to 6.95] | 5.48 [5.26 to 5.69] | 6.05 [5.87 to 6.23] | 6.82 [6.57 to 7.07] |
| 2007 | 6.58 [6.33 to 6.83] | 5.41 [5.19 to 5.62] | 6.03 [5.85 to 6.20] | 6.54 [6.30 to 6.79] |
| 2008 | 6.53 [6.28 to 6.77] | 5.22 [5.01 to 5.43] | 5.95 [5.78 to 6.13] | 6.15 [5.92 to 6.38] |
| 2009 | 6.18 [5.94 to 6.42] | 5.09 [4.89 to 5.30] | 5.61 [5.44 to 5.78] | 6.10 [5.87 to 6.33] |
| 2010 | 5.97 [5.73 to 6.20] | 4.94 [4.74 to 5.14] | 5.80 [5.63 to 5.97] | 6.26 [6.03 to 6.50] |
| 2011 | 5.51 [5.28 to 5.73] | 4.89 [4.70 to 5.09] | 5.50 [5.34 to 5.67] | 5.86 [5.64 to 6.08] |
| 2012 | 5.76 [5.53 to 5.99] | 4.66 [4.47 to 4.85] | 5.36 [5.20 to 5.52] | 5.80 [5.59 to 6.02] |
| 2013 | 5.47 [5.25 to 5.69] | 4.49 [4.30 to 4.68] | 5.44 [5.28 to 5.60] | 5.59 [5.38 to 5.80] |
| 2014 | 5.51 [5.29 to 5.74] | 4.44 [4.25 to 4.62] | 5.21 [5.05 to 5.36] | 5.74 [5.53 to 5.95] |
| 2015 | 5.18 [4.97 to 5.40] | 4.55 [4.37 to 4.74] | 5.17 [5.02 to 5.32] | 5.36 [5.16 to 5.56] |
| 2016 | 5.24 [5.02 to 5.45] | 4.31 [4.13 to 4.49] | 5.05 [4.90 to 5.20] | 5.60 [5.40 to 5.81] |
| 2017 | 5.04 [4.83 to 5.25] | 4.04 [3.86 to 4.21] | 4.89 [4.75 to 5.04] | 5.26 [5.06 to 5.45] |
| 2018 | 4.73 [4.53 to 4.93] | 4.06 [3.89 to 4.24] | 4.76 [4.62 to 4.90] | 5.22 [5.03 to 5.41] |
| 2019 | 4.78 [4.58 to 4.98] | 4.01 [3.84 to 4.18] | 4.69 [4.55 to 4.83] | 5.02 [4.83 to 5.20] |
| 2020 | 4.69 [4.49 to 4.89] | 3.92 [3.75 to 4.09] | 4.76 [4.63 to 4.90] | 5.21 [5.02 to 5.39] |

**Supplementary Table 5. Age-adjusted mortality rate due to Gastrointestinal cancer in the U.S stratified by census region.**

| **Year** | **Age Adjusted Mortality Rate (95% CI)** | |
| --- | --- | --- |
|  | **Metropolitan (Urban)** | **Non-metropolitan (Rural)** |
| 1999 | 8.26 (8.11 to 8.41) | 6.71 (6.43 to 6.99) |
| 2000 | 8.01 (7.87 to 8.16) | 6.73 (6.45 to 7.01) |
| 2001 | 7.69 (7.55 to 7.83) | 6.14 (5.88 to 6.41) |
| 2002 | 7.44 (7.30 to 7.58) | 5.97 (5.71 to 6.24) |
| 2003 | 7.17 (7.04 to 7.30) | 6.25 (5.98 to 6.52) |
| 2004 | 6.97 (6.83 to 7.10) | 5.74 (5.48 to 5.99) |
| 2005 | 6.66 (6.54 to 6.79) | 5.46 (5.21 to 5.70) |
| 2006 | 6.36 (6.24 to 6.48) | 5.45 (5.21 to 5.70) |
| 2007 | 6.33 (6.21 to 6.45) | 5.13 (4.89 to 5.37) |
| 2008 | 6.11 (5.99 to 6.23) | 5.17 (4.94 to 5.41) |
| 2009 | 5.90 (5.78 to 6.01) | 4.94 (4.71 to 5.17) |
| 2010 | 5.89 (5.78 to 6.01) | 5.08 (4.85 to 5.32) |
| 2011 | 5.65 (5.54 to 5.76) | 4.60 (4.38 to 4.82) |
| 2012 | 5.55 (5.44 to 5.66) | 4.59 (4.37 to 4.81) |
| 2013 | 5.41 (5.30 to 5.52) | 4.70 (4.49 to 4.92) |
| 2014 | 5.34 (5.24 to 5.45) | 4.58 (4.36 to 4.79) |
| 2015 | 5.21 (5.11 to 5.31) | 4.41 (4.20 to 4.62) |
| 2016 | 5.18 (5.08 to 5.28) | 4.38 (4.17 to 4.59) |
| 2017 | 4.91 (4.81 to 5.00) | 4.36 (4.15 to 4.57) |
| 2018 | 4.82 (4.72 to 4.91) | 4.13 (3.93 to 4.33) |
| 2019 | 4.71 (4.61 to 4.80) | 4.12 (3.92 to 4.32) |
| 2020 | 4.77 (4.68 to 4.86) | 4.15 (3.95 to 4.34) |

**Supplementary Table 6. Age-adjusted mortality rate due to Gastrointestinal cancer in the U.S stratified by urban-rural status.**
